# Genotypic and phenotypic characterization of G6PD deficiency in Bengali adults with severe and uncomplicated malaria

**DOI:** 10.1186/s12936-017-1788-x

**Published:** 2017-03-29

**Authors:** Katherine Plewes, Ingfar Soontarawirat, Aniruddha Ghose, Germana Bancone, Hugh W. F. Kingston, M. Trent Herdman, Stije J. Leopold, Haruhiko Ishioka, Md. Abul Faiz, Nicholas M. Anstey, Nicholas P. J. Day, Md. Amir Hossain, Mallika Imwong, Arjen M. Dondorp, Charles J. Woodrow

**Affiliations:** 1 Mahidol Oxford Tropical Medicine Research Unit, Faculty of Tropical Medicine, Mahidol University, 420/6 Rajvithi Road, Rajthevee, Bangkok, 10400 Thailand; 2 Centre for Tropical Medicine and Global Health, Nuffield Department of Medicine, University of Oxford, Oxford, UK; 3 Department of Clinical Tropical Medicine, Faculty of Tropical Medicine, Mahidol University, Bangkok, Thailand; 4 Department of Medicine, Chittagong Medical College Hospital, Chittagong, Bangladesh; 5 Shoklo Malaria Research Unit, Mahidol Oxford Tropical Medicine Research Unit, Faculty of Tropical Medicine, Mahidol University, Mae Sot, Thailand; 6 Global and Tropical Health Division, Menzies School of Health Research, Charles Darwin University, Darwin, NT Australia; 7 Malaria Research Group, and Dev Care Foundation, Dhaka, Bangladesh; 8 Department of Molecular Tropical Medicine and Genetics, Faculty of Tropical Medicine, Mahidol University, Bangkok, Thailand

**Keywords:** Glucose-6-phosphate dehydrogenase deficiency, Bangladesh, Falciparum malaria, Genotype

## Abstract

**Background:**

Control of malaria increasingly involves administration of 8-aminoquinolines, with accompanying risk of haemolysis in individuals with glucose-6-phosphate dehydrogenase (G6PD) deficiency. Few data on the prevalence and genotypic basis of G6PD deficiency are available from Bangladesh, where malaria remains a major problem in the South (Chittagong Division). The aim of this study was to determine the prevalence of G6PD deficiency, and associated G6PD genotypes, in adults with falciparum malaria in southern Bangladesh.

**Methods:**

G6PD status was assessed via a combination of fluorescent spot testing (FST) and genotyping in 141 Bengali patients admitted with falciparum malaria to two centres in Chittagong Division from 2012 to 2014. In addition, an analysis of genomic data from 1000 Genomes Project was carried out among five healthy Indian subcontinent populations.

**Results:**

One male patient with uncomplicated malaria was found to have G6PD deficiency on FST and a genotype associated with deficiency (hemizygous Orissa variant). In addition, there were two female patients heterozygous for deficiency variants (Orissa and Kerala-Kalyan). These three patients had a relatively long duration of symptoms prior to admission compared to G6PD normal cases, possibly suggesting an interaction with parasite multiplication rate. In addition, one of 27 healthy local controls was deficient on FST and hemizygous for the Mahidol variant of G6PD deficiency. Examination of 1000 Genomes Project sequencing data across the Indian subcontinent showed that 19/723 chromosomes (2.63%) carried a variant associated with deficiency. In the Bengali from Bangladesh 1000 Genomes population, three of 130 chromosomes (2.31%) carried deficient alleles; this included single chromosomes carrying the Kerala-Kalyan and Orissa variants.

**Conclusions:**

In line with other recent work, G6PD deficiency is uncommon in Bengalis in Bangladesh. Further studies of particular ethnic groups are needed to evaluate the potential risk of wide deployment of primaquine in malaria control efforts in Bangladesh.

## Background

Glucose-6-phosphate dehydrogenase (G6PD) deficiency is an X-linked enzyme deficiency present in more than 400 million people worldwide [1]. The similar global distribution of G6PD deficiency and malaria endemicity led to the hypothesis that this mutation confers protection against malaria [2, 3]. Evidence from large case control studies indicates that G6PD deficiency protects against cerebral malaria [4–7] and against high parasitaemias [8, 9]. The mechanism of protection and the fate of infected G6PD deficient red blood cells (RBCs) are unclear.

The coincident distribution of G6PD deficiency and malaria leads to a practical problem since the 8-aminoquinoline primaquine, the only drug active against hypnozoites used as radical treatment of vivax malaria, can cause severe haemolysis in G6PD deficient individuals [10]. Thus, determining the prevalence of G6PD deficiency in malaria endemic countries is critical with respect to malaria control and elimination programmes [11].

In Bangladesh, approximately 14 million people are at risk for malaria, with over 85% of cases occurring in Chittagong Division (80% in the Chittagong Hill Tracts) [12]. At the outset of this study there was a paucity of literature on the proportion of healthy individuals or malaria patients with G6PD deficiency in Bangladesh. Among 150 Bengali males attending an outpatient clinic or admitted to hospital in Dhaka, 4% were found to have enzymatic deficiency [13]. Prior to the commencement of this study, there were no data on prevalence of G6PD deficiency in Chittagong Division or malaria patients anywhere in the country, and no G6PD genotypic data of any form. The aim of this study was to determine the prevalence of G6PD deficiency and associated G6PD deficient genotypes in adults with falciparum malaria presenting at two locations in Chittagong Division.

## Methods

### Study site

The study was undertaken at two sites in southern Bangladesh (Chittagong Division) as part of a prospective observational study assessing the pathophysiology of falciparum malaria and a randomized controlled trial of paracetamol in severe and moderately severe malaria from 2012 to 2014. Ramu Upazila Health Complex is a rural centre in the District of Cox’s Bazar, ten miles east of Cox’s Bazar town, close to the Chittagong Hill Tracts and Myanmar border. Chittagong Medical College Hospital is an urban tertiary care centre in the city of Chittagong. Written, informed consent was obtained from all patients or attending relatives. Ethical approval was obtained from the Oxford Tropical Research Ethics Committee and Chittagong Medical College Hospital. ClinicalTrials.gov registration: NCT01641289.

### Patients

Patients admitted with asexual stage *P. falciparum* slide confirmed malaria were enrolled consecutively upon diagnosis. Criteria for severe malaria were: coma (Glasgow Coma Score <11), shock [systolic blood pressure (SBP) <80 mmHg with cool extremities], anaemia, (haematocrit <20% plus parasitaemia >100,000/µl), jaundice (total bilirubin >51.3 μmol/l plus parasitaemia >100,000/µl), hyperparasitaemia (>10%), acidosis (bicarbonate <15 mmol/l), hyperlactataemia (lactate >4 mmol/l), hypoglycaemia (glucose <2.22 mmol/l), convulsions (≥2/24 h), pulmonary oedema, and/or AKI (creatinine >265 μmol/l). Uncomplicated malaria was defined as slide positive malaria without severity criteria. Patients were managed according to WHO treatment guidelines [14]. Antimalarial treatment with parenteral artesunate (Guilin No. 2, China) was promptly administered at diagnosis followed by artemether/lumefantrine (Novartis, Switzerland) to complete therapy once oral medication was tolerated. A control group of healthy controls (n = 27) among local hospital staff was also recruited in Chittagong.

### Study procedures and assays

Detailed history and physical examination were performed after enrolment. Venous blood samples were drawn on enrolment for electrolyte, glucose, pH, lactate, and bicarbonate quantification using a handheld bedside analyzer (iSTAT, Abbott). Parasitaemia was assessed from thick and thin smears 6-hourly until parasite clearance. Plasma samples were also frozen in liquid nitrogen for subsequent analysis including measurement of *Plasmodium falciparum* histidine rich protein 2 (PfHRP2) and cell-free haemoglobin concentrations by ELISA as previously described [15, 16].

G6PD deficiency was defined by a combination of phenotyping and genotyping. Phenotyping was undertaken using the G6PD rapid fluorescent spot test (FST) (R&D Diagnostics, Greece) at enrolment and 4-week follow up (if the patient attended). Results were classified as ‘normal’, ‘intermediate’ and ‘deficient’. Intermediate classification was assigned if there was fluorescence but less intense than that observed with the G6PD normal control.

All patients enrolled into the paracetamol clinical trial, as well as any other malaria patients or healthy controls with an intermediate or deficient result by FST, had G6PD genotyping performed (n = 78). DNA for G6PD genotyping was extracted from frozen EDTA blood samples using the QIAamp^®^ DNA Mini Kit (QIAGEN, Germany) as per manufacturer’s instructions. Eluted genomic DNA from all samples was stored at −20 °C until polymerase chain amplification (PCR). G6PD gene sequencing was performed with seven pairs of primers designed to amplify all G6PD coding regions for direct DNA sequencing [17–19]. The two silent polymorphic markers, 1311C>T [20] and IVSXI C93T [21], were assessed by amplification of exon 11 and 12 along with introns 11 and 12, as previously described [19, 22]. DNA sequences were assessed by direct sequencing of the PCR product (Macrogen, Seoul, Korea) and analysed with BioEdit bioinformatics programme [365] using NCBI Reference Sequence X55448.1 as G6PD reference.

G6PD deficiency was defined as cases with deficiency on FST combined with a genotype compatible with deficiency (hemizygous, heterozygous or homozygous). The rationale for requiring genotypic as well as phenotypic evidence was that for rare conditions the positive predictive value of a test, even if it is highly specific [23], is low, leading to a high proportion of false positives (false deficients).

### 1000 Genomes data

G6PD genotypes and haplotypes for the five populations from the Indian subcontinent were downloaded from the expanded 1000 Genomes Project [24] and the frequency of mutations and prevalence of haplotypes at the G6PD marker polymorphisms 1311C**>**T and IVSXI C93T determined using chromosomes as the denominator.

## Results

### Recruitment

**Fig. 1:**
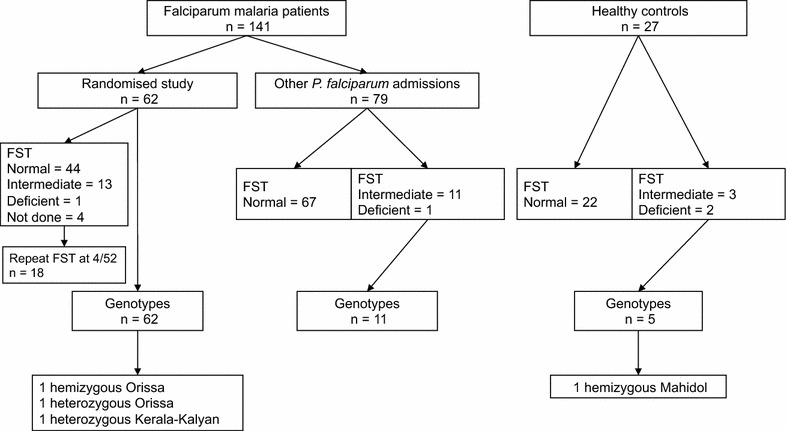
Study flow

During the study period 141 falciparum malaria patients had their G6PD status assessed by FST and/or genotyping (Fig. 1). Ninety-two (65.2%) were males and the mean (SD) age was 32 (14). All subjects were of Bengali ethnicity.

### G6PD phenotyping

The enrolment FST showed G6PD deficiency in 2/137 (1.5%) patients (both males) and intermediate results in 24/137 (17.5%) (11 females, 13 males) (Table 1). 18 (13%) patients returned for a repeat FST at 4 weeks; 12/18 results were the same at both time points (one deficient, one intermediate, ten normal) while six intermediate results on enrolment had a normal G6PD result at follow up (two males, four females).

**Table 1:** Results of G6PD fluorescent spot test

Result	SM (n = 75)	UM (n = 62)	Healthy (n = 27)
Deficient	1 (1.3%) 1M	1 (1.6%) 1M	2 (7.4%) 1M/1F
Intermediate	10 (13.3%) 5M/5F	14 (22.6%) 8M/6F	3 (11.1%) 3M/0F
Normal	64 (85.3%) 42M/22F	47 (75.8%) 33M/14F	22 (81.5%) 13M/9F

*SM* severe malaria, *UM* uncomplicated malaria, *M* male, *F* female

In the same period 27 healthy controls were recruited and underwent assessment by FST. Two (7%) were G6PD deficient (one male and one female).

Across all initial FST assessments, individuals with intermediate results had a significantly higher haemoglobin concentration at baseline than patients with a normal G6PD results (median 11.6 vs. 10.3 g/dl haemoglobin; p = 0.044).

### G6PD genotyping

**Fig. 2:**
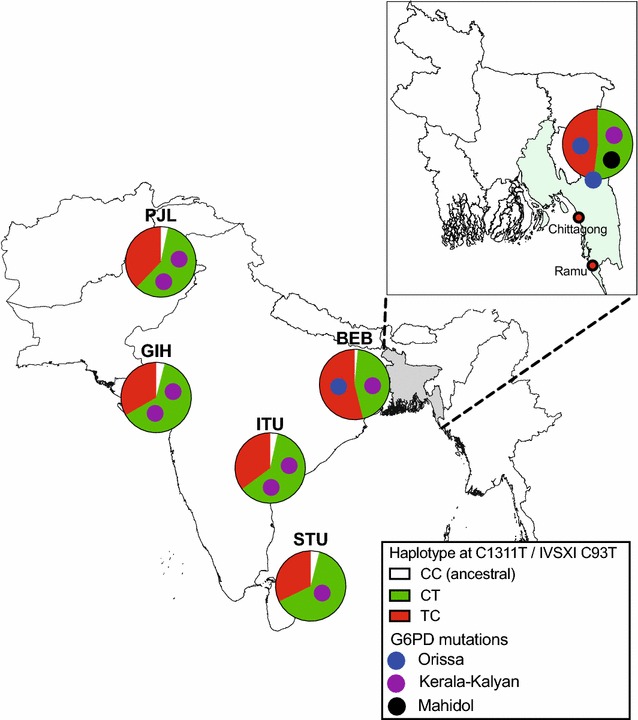
Proportions of haplotypes at the G6PD marker polymorphisms 1311C>T and IVSXI C93T in South Asian individuals in the 1000 Genomes Project, along with analogous data from this study in Bangladesh (*inset*). *Small circles* within pies represent individual chromosomes bearing one of the mutations found in this study (see key), with placement within each pie chart indicating the haplotype background. Population abbreviations: *BEB* Bengali from Bangladesh, *GIH* Gujarati Indian from Houston, *ITU* Indian Telugu from UK, *PJL* Punjabi from Lahore, *STU* Sri Lankan Tamil from the UK

Across the 78 individuals in whom G6PD was sequenced, 488 out of 546 PCR reactions produced clear sequence (89.4%). A non-synonymous G6PD mutation known to be associated with deficiency was found in three malaria patients (4.8%), all recruited within the randomized study (Figs. 1, 2). Two patients had the Orissa variant (131C>G); one was a hemizygous male (deficient on FST) and the other was a heterozygous female in whom the FST had not been performed. The third patient was heterozygous for the Kerala-Kalyan variant (949G>A) and had an intermediate FST on enrolment but normal FST at 4-week follow up (Table 2). A fourth male patient had a synonymous mutation in exon 7 (690C>T) and an intermediate FST. This mutation has not been published in the literature or the Leiden Open Variation Database [25, 26] and thus may be unique to Bangladesh.

**Table 2:** G6PD genotypes and markers identified in this study

Mutation	Variant	Location	Ancestral allele	Derived allele	Amino acid effect	Enzyme activity
c.131C>G	Orissa	Exon 3	C	G	A44G	~10% normal activity [37]
c.487G>A	Mahidol	Exon 6	G	A	G163S	<10% normal activity [38]
c.949G>A	Kerala-Kalyan	Exon 9	G	A	E317K	~20% normal activity [37]
c.690C>T	Unknown	Exon 7	C	T	I230I	Normal
c.1311C>T	–	Exon11	C	T	Y437Y	Normal
IVSXI 93C>T	–	Intron 11	C	T	Non-coding	Presumed normal

Of the two healthy controls with a deficient FST result, one male patient was found to have the Mahidol mutation (487G>A).

One male patient with severe malaria and one female control had a deficient FST but no mutation at any of the exons sequenced, indicating a false deficient FST. Alternative explanations include a polymorphism in the promoter or a splicing site [27].

### Polymorphic markers in G6PD

Two polymorphic markers in G6PD were also examined: the synonymous coding sequence 1311C>T [20, 28] and the non-coding polymorphism in exon 11, IVSXI C93T [21], both of which are thought to be of no functional importance (Table 2). The CC haplotype is the ancestral haplotype [22]. From the 78 genotyped individuals, 104 chromosomes were successfully genotyped at both markers with 44 having the CT haplotype and 40 the TC haplotype while 20 (from 10 females) were heterozygous at both markers preventing firm assignation of a haplotype. On the assumption that all ten carried one CT and TC haplotype each (since the TT haplotype involving both mutations has never been described), 54 chromosomes had the CT haplotype and 50 the TC haplotype (Table 3).

**Table 3:** Numbers of chromosomes with each of ten haplotypes found in the 1000 Genomes Database (Indian subcontinent populations) and this study

Orissa	Gond	Mahidol	Mediterranean	Coimbra	Kerala-Kalyan	C1311T	IVSXI C93T	BEB	GIH	ITU	PJL	STU	This study
C	G	G	C	C	G	C	C	2	7	5	5	6	0
C	G	G	C	C	G	**T**	C	69	50	51	56	47	50
C	G	G	C	C	G	C	**T**	56	92	86	82	90	54
**G**	G	G	C	C	G	**T**	C	1					1 (Case 1)^a^
C	**C**	G	C	C	G	C	**T**			1	1	3	
C	**C**	G	C	C	G	**T**	C					1	
C	G	**A**	C	C	G	C	**T**						1 (HC)
C	G	G	**T**	C	G	C	**T**				2	1	
C	G	G	C	**T**	G	C	**T**	1					
C	G	G	C	C	**A**	C	**T**	1	2	2	2	1	1 (Case 3)

Mutant alleles are shown in bold letters

*BEB* Bengali from Bangladesh, *GIH* Gujarati Indian from Houston, *ITU* Indian Telugu from UK, *PJL* Punjabi from Lahore, *STU* Sri Lankan Tamil from the UK

^a^In one patient with heterozygous Orissa mutation (Case 2) the haplotype could not be determined due to compound heterozygosity at both the marker polymorphisms C1311T and IVSXI C93T

The Mahidol (hemizygous) and Kerala-Kalyan (heterozygous) variants were associated with the CT haplotype while the Orissa variant (hemizygous) was associated with the TC haplotype (Table 3; Fig. 2). The heterozygous female with the Orissa variant was also heterozygous at both polymorphic markers preventing confirmation of background haplotype.

### 1000 Genomes data

Analysis of five 1000 Genomes Project populations from the Indian subcontinent (a total of 723 chromosomes) showed that G6PD deficiency genotypes were relatively rare (19 chromosomes overall = 2.63%) (Table 3; Fig. 2). The Kerala-Kalyan variant was the most common deficiency mutation, being found at low levels in all five populations (eight chromosomes overall). Kerala-Kalyan, Orissa and Coimbra variants were each present in a single chromosome in the Bengali from Bangladesh (BEB) group (total 130 chromosomes). Two other functional mutations (Gond and Mediterranean) were encountered in the wider Indian subcontinent (Table 3), being present on 5 and 3 chromosomes, respectively. The Mahidol variant was not present in any sample in the 1000 Genomes Indian subcontinent dataset.

Four of the Indian subcontinent populations had a predominance of the CT polymorphic marker haplotype consistent with previous results [22], while the BEB group showed a broadly similar prevalence of the two backgrounds. For deficiency alleles found in both this study and 1000 Genomes Project, the background haplotypes were the same in both studies i.e. CT for the Kerala-Kalyan variant and TC for Orissa (Table 3; Fig. 2).

### Clinical characteristics of malaria patients with G6PD deficiency genotypes

Clinical data for the three malaria cases with evidence of G6PD deficiency mutations were examined (Table 4). Parasite density is difficult to interpret since many patients reported receiving antimalarials prior to enrolment. The duration of illness (days of fever) among patients with a G6PD deficient variant was longer than the median duration in G6PD normal patients. Despite this, there was a trend to lower *Pf*HRP2 in G6PD deficient patients. There was also a trend to lower cell-free haemoglobin on enrolment in the G6PD deficient patients [median, range: 1.4 μM (0.9–1.6 μM) versus 3.1 μM (1.6–9.3 μM), p = 0.08]. None of the G6PD deficient patients had visible haemoglobinuria on enrolment or during admission. Of the G6PD normal patients that had haemoglobinuria (n = 18), the median cell-free haemoglobin concentration on admission was 14.7 μM (10.7–40.8 μM).

**Table 4:** Clinical characteristics of falciparum malaria patients

	Case 1	Case 2	Case 3	G6PD normal				
				SM (n = 77)	n	UM (n = 61)	n	
G6PD variant	Orissa	Orissa^b^	Kerala-Kalyan^b^	–		–		
Florescent spot test 0 h	Deficient	ND	Intermediate	64 N/10 I/1 D	75	47 N/13 I	60	
Demographics								
Age (years)	27	32	55	28 (22–40)	77	28 (21–35)	61	0.8468
Sex (%, M)	M	F	F	49 (64%)	77	42 (69%)	61	
Previous malaria	Y	N	N	16 (21%)	76	21 (34%)	61	0.191
Days of fever	10	13	30	7 (6–10)	77	7 (4–10)	61	0.0656
Blackwater fever	N	N	N	16 (21%)	77	2 (3%)	61	<0.0001
Severity of disease	UM	SM	UM	–	–	–	–	
Physical examination								
Temperature (°C)	40.0	36.8	37.0	38.2 (37.5–39.2)	77	38.2 (37.2–39.4)	61	0.89
Glasgow coma score	15	15	15	9 (8–14)	77	15 (15–15)	61	
Respiratory rate (per min)	40	32	28	36 (28–44)	77	26 (22–32)	61	<0.00001
Splenomegaly	N	N	N	16 (21%)	77	7 (11%)	61	N = 37/33
Laboratory parameters								
Parasite count per μl^a^	171,821	10,425	2520	100,229 (15,826–353,690)	77	22,244 (1200–102,615)	61	0.0006
*Pf*HRP2 (mg/ml)	384	1297	172	3036 (1497–9946)	71	563 (143–1300)	57	<0.0001
CFH (μM)	1.4	1.6	0.9	5.3 (2.5–12.7)	75	2.0 (1.3–3.6)	57	<0.0001
Haemoglobin (g/dl)	10.9	5.3	7.8	8.9 (7.0–10.9)	77	11.6 (8.9–13.2)	61	<0.0001
LDH (U/l)	141	967	275	643 (526–912)	77	322 (230–383)	28	<0.0001
Total bilirubin (mg/dl)^a^	1.4	1.4	0.6	2.0 (1.0–3.3)	77	1.0 (0.7–1.6)	60	0.0001
Creatinine (mg/dl)	1.1	4.5	1.1	1.4 (1.1–2.9)	77	1.2 (1.0–1.4)	60	0.0002
Urine pH	5.06	5.00	6.45	5.57 (5.28–5.83)	57	6.00 (5.62–6.43)	36	0.0004
pH	7.47	7.37	7.39	7.38 (7.32–7.43)	77	7.44 (7.40–7.47)	61	<0.0001
Bicarbonate (mmol/l)	19.8	13.9	22.6	17.3 (14.6–19.5)	77	21.4 (19.6–23.5)	61	<0.0001
pCO_2_ (mmHg)	28	24	37	29 (25–33)	77	32 (28–35)	61	0.0024
Lactate (mmol/l)	2.11	0.71	1.94	3.9 (2.3–6.1)	77	1.6 (1.2–2.1)	61	<0.0001

G6PD normal patients were either normal on FST or had normal G6PD sequence results

Data are number (%) or median (IQR), unless otherwise indicated

*M* male, *F* female, *Y* yes, *N* no, *UM* uncomplicated malaria, *SM* severe malaria

^a^Geometric mean (95% CI)

^b^Heterozygous

Case 1 was a 27-year old male with G6PD deficiency Orissa variant and uncomplicated malaria. Haemoglobinuria was absent on admission and did not develop during admission. In the absence of prior anti-malarial treatment, his parasitaemia was above the 75% percentile of G6PD normal uncomplicated malaria patients, which was accompanied by an elevated *Pf*HRP2 concentration. The cell-free haemoglobin was lower on enrolment compared to G6PD normal uncomplicated malaria patients and exhibited a bimodal pattern with a peak of 6.0 μM 42 h after G6PD normal patients. The creatinine was normal on admission and he did not develop kidney dysfunction. His recovery was unremarkable although he presented again 6 weeks later with *Plasmodium vivax* infection.

Case 2 was a 32-year old female heterozygous for the Orissa mutation (FST not done) with severe malaria, acute kidney injury (creatinine >265 μmol/l), and acidosis (bicarbonate <15 mmol/l) presenting after 2 days of oral artemether/lumefantrine. She did not present with or develop blackwater fever but gave a history of red urine prior to admission and had haemoglobin in her urine by dipstick on enrolment and at 76 h. Anaemia was present on enrolment, requiring three blood transfusions during admission. The fractional excretion of sodium was >2% suggesting acute tubular injury, while the urine pH (<5.3) and urine anion gap suggested a type 2 renal tubular acidosis. Cell-free haemoglobin on enrolment was below the 75% percentile compared to G6PD normal severe malaria patients but increased to 6.7 μM at 72 h. Dialysis was initiated 6 h after enrolment and an additional 5 cycles were required. She was discharged 3 weeks after enrolment and kidney function returned to baseline at 6 weeks.

Case 3 was a 55-year-old female heterozygous for the Kerala-Kalyan mutation with uncomplicated malaria enrolled after three doses of intravenous quinine. Anaemia was present at enrolment but no blood transfusion was required. Haemoglobin was absent from the urine throughout. Cell-free haemoglobin was low on admission but increased to 16.2 μM at 24 h. Creatinine was normal on enrolment and acute kidney injury did not develop during admission. Although the FST was intermediate at admission, at 4-week follow-up a normal result was obtained.

## Discussion

This study found that G6PD deficiency was uncommon in Bengalis admitted to hospital with *P. falciparum* malaria in two centres in southern Bangladesh. One out of 141 (0.7%, 95% CI 0.04–3.9) *P. falciparum* patients was G6PD deficient on the basis of fluorescent spot testing followed by genotyping, while two patients were heterozygous at known deficiency alleles. A combination of phenotyping and genotyping was used for several reasons. Firstly, the FST accurately diagnoses severe G6PD deficiency [23] at activity levels below 30% of population median, but its positive predictive value is still likely to be low when the population prevalence of deficiency is low. Hence a second test to confirm the presence of a genotype compatible with deficiency was required. Secondly, consideration of only cases with clear deficiency on the FST was likely to be insensitive for detection of cases with intermediate levels of G6PD activity seen in a proportion of female heterozygotes [23, 29, 30]. For this reason, all cases with an intermediate result on the FST were genotyped. However, most cases with an intermediate result on the FST did not have genotypic evidence of deficiency. While it is theoretically possible that in these cases deficiency variants were missed because of incomplete sequence (approximately 10% of PCR reactions were unsuccessful), as a group these intermediate cases are likely to have been false deficients since most of the cases with intermediate FST results at enrolment who returned for follow-up had normal FST results at 4 weeks. Rarity of the underlying condition is also likely to have produced a relatively large number of false deficients (i.e. low positive predictive value).

One particular factor that might have led to intermediate FST results (but no genotypic evidence of G6PD deficiency) could have been the observed heterogeneity in FST appearances according to haemoglobin level. It was noted that the haemoglobin concentration was significantly higher among the intermediate group compared to the G6PD normal group; this is consistent with the observation that G6PD-normal anemic subjects (especially pregnant women) tend to show a brighter fluorescence compared with G6PD-normal subjects without anaemia [31]. This decreased fluorescence (intermediate FST) may be due to increased quenching effect of haemoglobin [32, 33] in patients with malaria.

Two studies of G6PD deficiency in Bangladesh were published during the conduct of our study. A phenotypic survey of G6PD deficiency in malaria patients undertaken in Bandarban Division, Chittagong Hill Tracts, Bangladesh [34], showed that 1/142 patients (0.7%) had severe G6PD deficiency (<10% of adjusted male median activity) and 5/142 (3.5%) had mild G6PD deficiency (10–60% of adjusted male median activity) [34]; most were male subjects. The overall prevalence of G6PD deficiency was in agreement with the work presented here.

In contrast, a study of randomly sampled individuals without malaria from the Marma and Khyang ethnic groups (n = 202, approximately 60% female) also living in the Chittagong Hill Tracts (CHT) showed that 59% had normal G6PD activity, 35% had mild deficiency and 6.5% had severe deficiency [35], with apparently higher levels of deficiency in the Marma population. These somewhat differing results might be explained by several factors. G6PD deficiency has been shown to be associated with lower parasitaemia in vivax malaria in the Karen population in Thailand [9], and protection against vivax malaria in the Pashtun population in Afghanistan where prevalence of G6PD deficiency is substantially lower in vivax patients than in the healthy population [36]; similar effects might be operating in Bangladesh with *P. falciparum*. However, 1000 Genomes Project data (based on complete G6PD genotypes from healthy subjects) also show a low prevalence of deficient genotypes in Bengalis from Bangladesh as well as several other populations from the Indian subcontinent. An alternative explanation is that the prevalence of deficiency may be significantly higher within relatively isolated ethnic groups owing to the forces of positive selection as well as genetic drift. Methodological differences in terms of blood storage and measurement might also explain the difference in prevalence. Further studies of the genotypic basis of G6PD deficiency in various ethnic groups in the Chittagong Hill Tracts are needed to shed further light on this area, and are indicated given the potential risk in these populations of haemolysis with pro-oxidant drugs such as primaquine (at the dose used for radical treatment of vivax malaria).

The three variants identified in this study were Orissa, Kerala-Kalyan, and Mahidol. While the study did not quantitatively assess G6PD deficiency, other studies have found these mutations to be associated with ~10% [37], ~20% [37] and <10% [38] normal enzymatic activity, respectively. Across the wider Indian subcontinent, the G6PD variants Mediterranean, Gond and Kerala-Kalyan are widely distributed (although not common in any location) while the Orissa variant is found predominantly in eastern and southern India [37, 39–44]. The Kerala-Kalyan variant has also been reported in individuals in southern Myanmar [18], Thailand (Phuket) and Mauritius [44]. The genetic marker backgrounds in this study were also consistent with previous studies; the Kerala-Kalyan variant was linked to the 1311C/IVSXI C93T haplotype background and the Orissa variant to the 1311C>T/IVSXI C93 background. The Mahidol variant (found in one healthy control in this study) has not been reported in India, and is the predominant variant across Myanmar [18, 40, 45, 46]. The overall proportion of haplotypes at the two polymorphic marker positions in patients without G6PD deficiency was also similar to 1000 Genomes Data from Bengalis in Bangladesh, supporting the genetic similarity between these populations [22].

Due to the low number of G6PD deficient malaria patients detected in this study, it was not possible to examine in any detail the potential interaction between the forms of deficiency found and the severity of falciparum malaria or its form of illness. Evidence largely gathered from Africa indicates that G6PD deficiency protects against cerebral malaria [4–7] and against high parasitaemias [8, 9], but relatively few data are available for Asia. Case 1 defined with G6PD deficiency had a relatively long duration of illness compared to G6PD normal cases, but relatively low total parasite burden (judged by plasma PfHRP2), likely reflecting overall low parasite multiplication rate, consistent with the possibly reduced falciparum parasite invasion [47] and growth [48] of G6PD deficient red cells observed in vitro. Interestingly, Case 2 presented with severe malaria with kidney injury, acidic urine, haemoglobinuria and metabolic acidosis with low plasma lactate. G6PD deficiency has been associated with haemoglobinuria and kidney dysfunction in adult and paediatric patients with and without malaria [49–56]. Experimental models suggest the urine free haemoglobin is nephrotoxic especially in the setting of dehydration [57], and aciduric urine [58], which converts haemoglobin to methaemoglobin [58]. Further, renal haptoglobin [59] and haemopexin [60] expression are upregulated with resultant higher urinary concentrations in response to various models of haemoprotein-mediated kidney injury. In a case series of G6PD deficient Brazilian patients with *P. vivax* administered daily 0.5 mg/kg primaquine, among those with a creatinine measurement 5/16 (31%) developed acute kidney injury [61].

## Conclusion

G6PD deficiency is not common in Bengalis with falciparum malaria assessed in two centres near Chittagong Hill Tracts, Bangladesh. One of 141 patients was deficient on FST screening and hemizygous for the Orissa G6PD variant, and there were also two heterozygote females (Orissa or Kerala-Kalyan variants), while a single case of hemizygous Mahidol deficiency was found among healthy controls. The overall prevalence of deficient alleles was similar to that in a healthy population of Bengalis from Bangladesh studied in the 1000 Genomes Project, and other populations across the wider Indian subcontinent. Further studies are required to determine whether the studied variants have a protective effect against malaria infection, to determine the haemolytic risk in response to malaria infection and 8-aminoquinolines, and to explore further the prevalence and genetic basis of G6PD deficiency in other ethnic groups in Bangladesh.

## Abbreviations

BEB: Bengali from Bangladesh
FST: fluorescent spot test
G6PD: glucose-6-phosphate dehydrogenase deficiency
GIH: Gujarati Indian from Houston
ITU: Indian Telugu from UK
*Pf*HRP2: *Plasmodium falciparum* histidine rich protein 2
PJL: Punjabi from Lahore
SM: severe malaria
STU: Sri Lankan Tamil from the UK
UM: uncomplicated malaria
